# Impact of high energy oral nutritional supplements consumed in the late afternoon on appetite, energy intake and cardio-metabolic risk factors in females with lower BMI

**DOI:** 10.1038/s41430-021-01042-w

**Published:** 2021-11-12

**Authors:** Sadia Fatima, Konstantinos Gerasimidis, Charlotte Wright, Dalia Malkova

**Affiliations:** 1 Human Nutrition, School of Medicine, Dentistry and Nursing, College of Medical, Veterinary and Life Sciences, University of Glasgow, Glasgow, UK; 2 Khyber Medical University Peshawar, Peshawar, Pakistan

**Keywords:** Physiology, Biomarkers

## Abstract

**Background/Objective:**

Morning consumption of a single dose of high-energy oral nutritional supplement (ONS) in females with a lower BMI displaces some of the food eaten at breakfast but increases overall daily energy intake. This study investigated the effectiveness of ONS intake in the late afternoon and for longer duration.

**Subjects/Methods:**

Twenty-one healthy females (mean ± SD, age 25 ± 5 years; BMI 18.7 ± 1.2 kg/m^2^) participated in a randomised, crossover study with two experimental trials. In the afternoon of days 1–5, participants consumed either ONS (2.510 MJ) or low-energy PLACEBO drink (0.377 MJ) and recorded food eaten at home. On day six, energy intake was measured during buffet meals, and energy expenditure, appetite measurements and blood samples were collected throughout the day.

**Result:**

Over the 5-day period, in the ONS trial energy intake from evening meals was lower (ONS, 2.7 ± 0.25 MJ; Placebo, 3.6 ± 0.25 MJ, *P* = 0.01) but averaged total daily energy intake was higher (ONS, 9.2 ± 0.3 MJ; PLACEBO, 8.2 ± 0.4 MJ, *P* = 0.03). On day six, energy intake, appetite scores, plasma GLP-1 and PYY, and energy expenditure were not significantly different between the two trials but fasting insulin concentration and HOMA_IR_, were higher (*P* < 0.05) and insulin sensitivity score based on fasting insulin and TAG lower (*P* < 0.05) in ONS trial.

**Conclusion:**

Late afternoon consumption of ONS for five consecutive days by females with a lower BMI has only a partial and short-lived energy intake suppression and thus increases daily energy intake but reduces insulin sensitivity.

## Introduction

Disease-related malnutrition is common among patients admitted to hospitals and among outpatients [1–3]. Several studies have shown that high-energy oral nutritional supplements (ONS) can increase energy intake and thus improve clinical outcomes and quality of life in undernourished individuals [4–7]. Although a single dose of ONS typically increases the daily energy intake by an average of 1.57 MJ/d, their overall benefit is lower than expected [8, 9]. It seems that the effectiveness of ONS in increasing energy intake is compromised by the partial displacement of food from habitual meals [10] rather than low adherence to the ONS prescription [11]. Thus, experimental research on factors that determine the level of energy intake compensation is important in providing insight into the role of ONS in modulating energy intake in malnourished individuals.

Our previous laboratory-controlled study conducted in healthy, women with a lower BMI showed that high energy ONS, taken on a single occasion, prior to breakfast, displaced some of the food eaten at breakfast and had no impact on energy intake later during the day and thus increased net energy intake by half of the total amount of energy supplied [12]. In clinical practice, ONS are usually provided in the evening, rather than prior to breakfast, and prescribed over several weeks and months [13]. In general, it has been suggested that energy intake in the evening is less satiating than energy intake in the morning [14]. Studies of bedtime snacks have found variable degrees of compensatory reduction in energy intake and most of these studies were conducted on overweight and obese, individuals [15, 16]. Thus, the degree of energy intake compensation for ONS taken in the evening remains to be investigated.

An energy surplus is known to affect the postprandial responses of gastrointestinal appetite hormones [17]. Thus, measuring appetite-regulating hormones is important in providing a mechanistic understanding of the impact of high energy ONS on energy intake. Our previous study found no effect of a single dose of high energy ONS, consumed 60 minutes before breakfast, on subjective appetite measured pre-breakfast and pre-lunch. However, plasma concentrations of anorexigenic glucagon-like peptide1 (GLP-1) and peptide YY (PYY), the most widely studied gut appetite hormones [18], were significantly higher after oral intake of high energy ONS than after Placebo [12]. This dissociation between the subjective appetite measures and plasma concentrations of satiety hormones has been observed in some other studies [19–21], but the impact of a positive energy balance created by medium-term high energy ONS on these interactions has not yet been investigated.

This study aimed to investigate the impact of five days of supplementation with ONS in the late afternoon on energy intake from home meals and total daily energy intake, and subjective appetite scores, postprandial concentrations of plasma GLP-1 and PYY and energy expenditure measured under controlled laboratory conditions. Since a dietary energy surplus may have a detrimental impact on metabolic risk factors of cardiovascular disease [22, 23], this study also investigated whether five days of supplementation with ONS affected plasma concentration of lipids, insulin, and glucose differently from PLACEBO.

## Material and methods

### Participants and ethics approval

The study was promoted using an advertisement leaflet and word of mouth at the University of Glasgow campus and in other public places. Eligible participants were young healthy females with a BMI of 17–20 kg/m^2^ whose body weight had been stable for one month prior to study enrolment and with sufficient physical activity: at least 150 min of moderate-intensity or 75 min of vigorous-intensity physical activity weekly, or any equivalent combination of the two [24]. Participants with chronic illness, eating disorders or previous gastrointestinal operations were excluded. All the participants were non-smokers, not pregnant, had a regular menstrual cycle, were on no medication or nutritional supplement and were not following a special diet. Participants underwent a screening visit that included height, weight, FM, and FFM measurement, a detailed health screening, and indicated food preferences from a shortlist of common breakfast and lunch items. In addition, the palatability of the ONS and Placebo drinks was tested, and participants who disliked the taste of the supplements (*n* = 3) or felt sick after supplement intake (*n* = 1) were not enrolled. All participants gave written informed consent prior to commencing the study. The Research Ethics Committee of The College of Medical Veterinary and Life Sciences of the University of Glasgow approved the study. The study was registered at www.controlled-trials.com as ISRCTN12092733.

### Study design overview

**Fig. 1:**
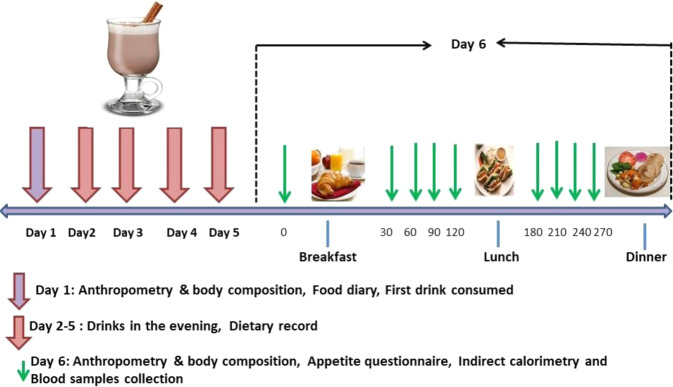
A schematic presentation of the study design during supplementation with ONS or PLACEBO on Day 1 - Day 5 and during Day 6 conducted under laboratory conditions.

This was a single-blinded, randomised, crossover study. Each participant underwent two experimental trials, one using a high-energy oral nutrition supplement (ONS) and the other with PLACEBO. Each trial lasted for 6 days, in a fully balanced design, with a washout of at least one week (Fig. 1). A block method of randomization was used to randomly allocate participants for taking the ONS or PLACEBO drink first. For each trial, in the evening of the five days, at ~6 pm, participants came to the metabolic suite and consumed either ONS (Scandishake, Chocolate, Nutricia) made up with 240 g of full-fat milk or PLACEBO (a low energy drink prepared with 240 g of skimmed milk, 4 g of cocoa and 2 g sweeteners). The drinks were volume, colour, texture and flavour matched but had different energy and macronutrient compositions (Supplementary Table 1).

On day 1, participants came to the metabolic suit ~4 hours after lunch intake. After consuming the drink, participants went home where they had their evening meal. All the timings for lunch, supplement and evening meal on day 1 of the first experimental trial were replicated on day 1 of the second experiment trial. During the following four days, participants kept a weighed record of all food and drink consumed at home, using food diaries and electronic food scales, with written instructions (Salter Housewares Ltd., Tonbridge, UK). During the first three days of the first trial, participants were asked to record the duration and intensity of any exercise sessions and to repeat this pattern of physical activity during the first three days of the second trial. On days four and five participants were asked to refrain from planned exercise and alcohol intake. On day 5 of each trial, the participants were asked to guess which type of drink they were taking.

On the morning of the day, six participants reported to the metabolic suite at 08:00–08:30 after ~12 hrs fast (Fig. 1). After anthropometry, participants filled in an appetite questionnaire, followed by resting energy expenditure measurements lasting for ~20 min. A venous cannula was then inserted, and a fasting blood sample was obtained 10 min later. Further appetite scores, blood samples and postprandial energy expenditure measurements were obtained at four-time points after *an ad libitum* buffet breakfast and at four-time points after lunch, with each measurement lasting for ~20 minutes. An *ad libitum* buffet dinner was then served seven hours after the breakfast.

### Anthropometry and body composition

Initial test-retest reliability was determined as described before [25]. Height, weight, BF, and FFM of the participants were measured wearing lightweight clothing immediately after urination, during the screening visit, on the first day of supplementation and the morning of day six. Height was measured with a portable stadiometer (Seca, Leicester, UK). Bodyweight, FM, and FFM were measured using a leg-to leg bioelectrical impedance scale (TBF-300, TANITA, Cranela, UK).

### Ad libitum buffet meals

The content of the breakfast and lunch buffets was determined based on the participant’s reported eating habits, excluding both top-rated foods and disliked ones to avoid over- and under- consumption. The meals served during both trials were identical (for full details see Supplementary method). Breakfast included a variety of breakfast cereals, milk, croissants, jam butter, fruit juice and whole fruits. Lunch included sandwiches, salad, yoghurt, fruit and juice. For dinner, participants selected the meal of their choice from the cafeteria of the local hospital, which was then served with fruits, yoghurt, and juice. Water was available, but participants were asked to consume the same amount at the same time in both trials. During meals, reading, listening to music and watching television were not allowed, as all these activities might influence food intake [26]. To eliminate portion-related cues, the food offered was cut into pieces. All the meals were served at the same time, on the same table, using the same dishes. The participants were given 30 minutes to consume their meals and advised to eat until comfortably full and satisfied. The researcher weighed all food and drinks offered and the left-overs of each meal with an electronic kitchen scale (Salter Housewares Ltd., Tonbridge, U.K.).

### Dietary intake analysis

Windiet 2005 (The Robert Gordon University, Aberdeen, Scotland, UK) was used to calculate the energy and macronutrient intake of all meals consumed. The dietary analysis was conducted by two researchers and the mean of their two estimates was used unless the difference in energy intake was >50 kcal. In this case, the dietary analysis was repeated. Intake records were excluded if the reported energy was greater than 2 * measured resting metabolic rate (RMR) (over-reporting) or less than 1.35 * RMR (under-reporting) [27].

### Appetite scores

Appetite was assessed using a validated Visual Analogue Scale (VAS) questionnaire (100 mm) [28]. The VAS questions were anchored with negative feeling words. Participants marked a horizontal line with “not at all” at one end and “extremely” at the other end at a point that most accurately reflected their feeling at that time of hunger, fullness, satiety, desire to eat and prospective food consumption.

### Energy expenditure

Rates of oxygen consumption (V̇O_2_) and carbon dioxide production (V̇CO_2_) were measured after each blood sample collection for the duration of 20 min by a computerised open-circuit ventilated hood system (Oxycon Pro, Jaeger GmbH, Germany) (see supplementary methods).

### Plasma preparation and blood analysis

For full details see supplementary methods. Venous blood samples were collected into EDTA tubes and the plasma used for the analysis of insulin and glucose and lipid concentrations. Because of their high cost, insulin, PYY and active GLP were measured in only a subset of 12 randomly selected participants. Glucose and lipids were also measured in 12 participants. The within-batch coefficients of variation were <3% for plasma lipids and glucose, <4% for the insulin and, < 8% for the activated GLP-1 and PYY assays.

### Power calculations and statistical analysis

Based on our previous publication [12] where the mean (SD) for energy intake was 1.07 (1.5) MJ higher after high-energy ONS than after Placebo, with a statistical power of 0.85 and probability error less than 0.05, we found that a minimum sample size of 18 participants would be necessary to detect a difference in energy intake of this size between ONS and PLACEBO trials.

Normally distributed variables (EI, fasting concentrations of plasma lipids, insulin, glucose, GLP-1 and PYY, and time-averaged areas under the variable curve (AUC) for appetite scores, concentrations of PYY, GLP-1, insulin, glucose and TAG were compared by paired t-test. Non-normally distributed variables (body weight, fat mass) were compared by the Mann-Whitney test. The appetite ratings, log-transformed plasma concentrations of active GLP-1 and PYY, insulin, glucose and TAG were analysed using two-way repeated-measures ANOVA, followed by a post hoc Tukey test. Trial order effects on energy intake during days of supplementation, energy intake on day 6, subjective appetite measures, appetite hormones, insulin, glucose and lipids were explored by ANOVA with an interaction term between the order of the trial and the effect of the latter (i.e., PLACEBO or ONS) being added in the General Linear Model. Statistical analysis was performed using Statistica (version 10.0; StatSoft, Inc., Tulsa, OK) and Minitab (version 17.3.1; Minitab, Inc., State College, PA).

Insulin resistance was calculated by the homeostasis model assessment (HOMA_IR_) [29] and insulin sensitivity was predicted from scores based on fasting insulin and fasting triglycerides [30]. Since insulin sensitivity can be calculated from meal tests [31], fasting, post-breakfast (0–120 min) and post-lunch (120–270 min) plasma glucose and insulin concentrations were used to calculate insulin sensitivity indexes (ISI) devised by Matsuda and DeFronzo [32].

## Results

### Participants

**Fig. 2:**
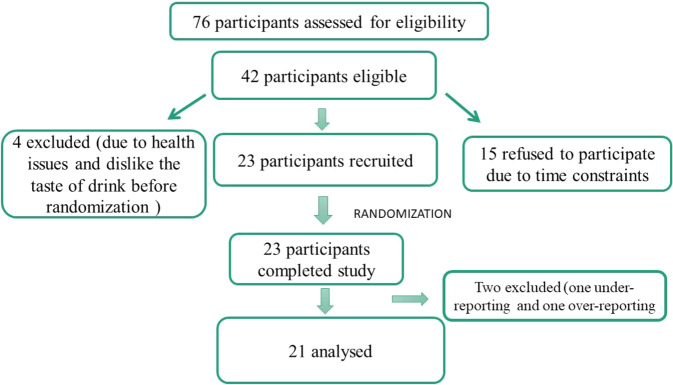
Flow chart of participant screening and recruitment.

Of 42 eligible participants, 15 declined to take part in the study because of time constraints and four were excluded before randomization due to dislike of the taste of the supplements and acute health issues Thus, 23 participants were randomised and completed the study (Fig. 2). Two participants were excluded from the final analysis, one because of under-reporting energy intake in the PLACEBO trial and another because of over-reporting energy intake in both trials. Thus, results are presented for 21 participants with a mean (SD) of 25.5 (5.3) years, the height of 1.65 (1.4) m, BMI of 18.7 (1.2) kg/m^2^, body fatness of 16.3 (1.03)%, FMI of 3.17 (1.04) kg/m^2^ and of FFMI of 15.64 (0.87) kg/m^2^_._

### Reported energy and macronutrient intake during supplementation days

Over four days of supplementation, reported daily averaged energy intake from meals eaten before taking the supplementation drinks was not different between the ONS and PLACEBO trials (Table 1) but energy intake at the evening meals and total daily energy intake calculated without inclusion of energy provided by ONS and PLACEBO drinks were significantly lower in the ONS than PLACEBO trial (Table 1). In the ONS trial, the reduction in energy intake during the evening meal was equivalent to 43% of the energy provided by the supplement. With the addition of energy and macronutrients provided by supplements, the reported total daily energy, carbohydrate and fat intake were significantly higher in the ONS than in the PLACEBO trial (Table 1). The extra energy in ONS trial was supplied by a 20% increase in carbohydrate and a 36% increase in fat intake.

**Table 1:** Reported energy intake (EI) and macronutrient intake during 4 days of supplementation in ONS and PLACEBO trials. Values are presented as Mean ± SE (*n* = 21).

		ONS	PLACEBO	*P*-value
Before Drink	EI (MJ)	4.0 ± 0.2	4.2 ± 0.2	0.43
	Carbohydrate (g)	192 ± 11	190 ± 14	0.86
	Protein (g)	50 ± 2	57 ± 4	0.08
	Fat (g)	53 ± 5	53 ± 5	0.97
After Drink^a^	EI (MJ)	2.8 ± 0.2	3.6 ± 0.2	0.02
	Carbohydrate (g)	25 ± 3	44.7 ± 4	0.00
	Protein (g)	6.9 ± 1.2	12.8 ± 0.7	0.00
	Fat (g)	7.8 ± 1.3	16.4 ± 3.5	0.01
Total Daily^a^	EI (MJ)	6.8 ± 0.3	7.7 ± 0.3	0.04
	Carbohydrate (g)	217 ± 11	237 ± 13	0.21
	Protein (g)	57 ± 3	70 ± 4	0.00
	Fat (g)	61 ± 6	70 ± 4	0.2
Total Daily^b^	EI (MJ)	9.2 ± 0.3	8.2 ± 0.4	0.03
	Carbohydrate (g)	286 ± 11	249 ± 13	0.03
	Protein (g)	69 ± 3	79 ± 4	0.02
	Fat (g)	91 ± 6	71 ± 4	0.01

*P* values (paired *t*-test) are for the difference between ONS and PLACEBO trials,

^a^without, ^b^with energy and macronutrients of ONS and PLACEBO Supplements.

### Bodyweight and body fatness

There was no difference in the change of body weight or fat mass from day one to day 6 (mean difference 0.1 kg, 95% confidence interval −0.30 to 0.40) or fat mass (mean difference 0.4 kg, 95% confidence interval −0.50 to 1.40) between the ONS and the PLACEBO.

### Responses after 5 days of supplementation

**Fig. 3:**
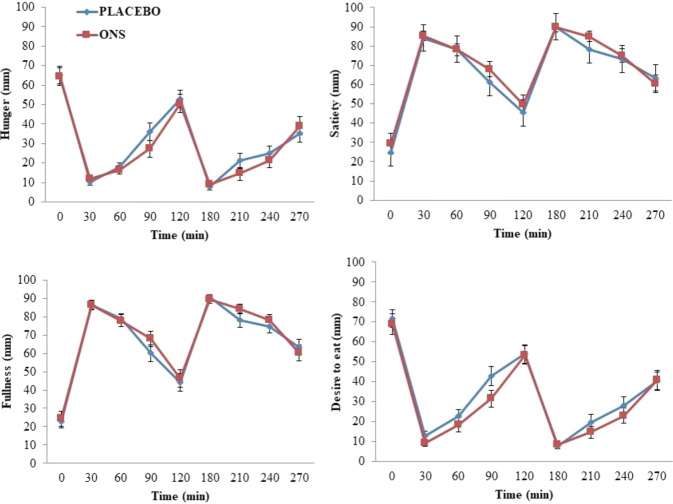
Fasting (0 min) and postprandial (0–270 min) responses of hunger, satiety, fullness, desire to eat, and prospective food consumption measured on the day following 5 days of supplementation with ONS and PLACEBO. *Ad libitum* breakfast and *ad libitum* lunch were provided after fasting measurements and 120 min after that, respectively. Values are presented as Means ± SE (*n* = 21). Responses were analysed by two-way repeated-measures ANOVA.

**Fig. 4:**
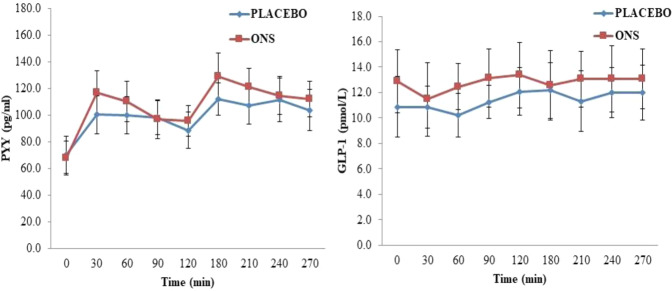
Fasting (0 min) and postprandial (0–270 min) plasma concentrations of peptide YY (PYY) and activated GLP-1 measured on the day following 5 days of supplementation with ONS and PLACEBO. *Ad libitum* breakfast and *ad libitum* lunch were provided after fasting measurements and at 120 min after that, respectively. Values are presented as Means ± SE (*n* = 12). Responses were analysed by two-way repeated-measures ANOVA.

There was no difference between the ONS and the PLACEBO trials in energy and macronutrient intakes during the meals consumed under laboratory conditions on day six (Table 2). The rate of resting and time-averaged post-breakfast and post-lunch energy expenditure was not different between ONS and PLACEBO trials (Supplementary Fig. 1). Subjective appetite ratings (Fig. 3) and plasma concentrations of GLP-1 and PYY (Fig. 4) were not different between trials. There was also no difference in time-averaged postprandial concentrations of GLP-1 and PYY after breakfast and lunch.

**Table 2:** Energy and macronutrient intake during *ad libitum* breakfast, lunch, and dinner consumed on the day following five days of supplementation with high energy ONS and PLACEBO drinks. Values are presented as Mean ± SE (*n* = 21).

Meal		ONS	PLACEBO	*P*-value
Breakfast	Energy Intake (MJ)	3.19 ± 0.21	3.19 ± 0.18	0.98
	CHO (g)	128 ± 7	129 ± 7	0.96
	Protein (g)	17 ± 1	17 ± 1	0.49
	Fat (g)	23 ± 3	24 ± 3	0.51
Lunch	Energy Intake (MJ)	2.65 ± 0.21	2.52 ± 0.19	0.54
	CHO (g)	81 ± 7	77 ± 6	0.53
	Protein (g)	28 ± 3	27 ± 3	0.71
	Fat (g)	24 ± 3	22 ± 2	0.45
Dinner	Energy Intake (MJ)	2.97 ± 0.24	2.90 ± 0.25	0.82
	CHO (g)	100 ± 10	97 ± 8	0.75
	Protein (g)	23 ± 2	22 ± 3	0.54
	Fat (g)	26 ± 3	27 ± 3	0.87
Total	Energy Intake (MJ)	8.93 ± 0.53	8.75 ± 0.49	0.69
	CHO (g)	310 ± 20	301 ± 15	0.57
	Protein (g)	68 ± 5	66 ± 5	0.65
	Fat (g)	72 ± 5	73 ± 6	0.96

*P* values (paired *t*-test) are for the difference between ONS and PLACEBO trials.

### Plasma lipids, glucose and insulin after 5 days of supplementation

After 5 days of supplementation, fasting insulin concentration and HOMA_IR_ were significantly higher and insulin sensitivity score based on fasting insulin and TAG significantly lower in the ONS than in the PLACEBO trial, and insulin sensitivity index by Matsuda calculated from fasting and post-breakfast (0–120 min) and post-lunch (120–270 min) plasma glucose and insulin concentrations showed a borderline difference (*P* = 0.08) between ONS and PLACEBO trials (Table 3, Supplementary Fig. 2). There was no difference in fasting concentrations of total-, HDL- and LDL-cholesterol, TAG and glucose, or postprandial concentrations of glucose, insulin and TAG (Table 3, Supplementary Fig. 2).

**Table 3:** Fasting concentrations, collected in a random subset of 12 participants, of plasma insulin, glucose, triglycerides (TAG), total-, HDL-, and LDL-cholesterol and time-averaged postprandial concentration of glucose, insulin, and TAG after 5 days of supplementation with high energy ONS and PLACEBO, and calculated HOMA_IR_, and insulin sensitivity indexes (ISI) for post-breakfast (0–120 min) and post-lunch (120–270 min) periods. Values are Mean ± SE.

	ONS	PLACEBO	*P*-value
Fasting Insulin (mU/L)	5.19 ± 0.56	3.47 ± 0.27	0.02
Fasting Glucose (mmol/L)	4.72 ± 0.19	4.55 ± 0.29	0.45
Fasting Total Cholesterol (mmol/L)	5.56 ± 1.13	5.30 ± 1.04	0.54
Fasting HDL-Cholesterol (mmol/L)	1.76 ± 0.31	1.62 ± 0.29	0.44
Fasting LDL-Cholesterol (mmol/L)	3.39 ± 0.84	3.23 ± 1.01	0.56
Fasting TAG (mmol/L)	0.88 ± 0.30	0.83 ± 0.27	0.47
Postprandial Insulin (mU/L)	24.22 ± 3.67	26.24 ± 4.11	0.42
Postprandial Glucose (mmol/L)	5.46 ± 0.21	5.51 ± 0.34	0.85
Postprandial TAG (mmol/L)	0.97 ± 0.35	0.99 ± 0.38	0.83
HOMA_IR_	1.08 ± 0.13	0.68 ± 0.05	0.01
Mffm/l	9.52 ± 0.38	10.81 ± 0.40	0.03
ISI (0–120 min)	12.0 ± 1.8	15.7 ± 2.1	0.08
ISI (120–270 min)	11.8 ± 1.5	14.3 ± 1.8	0.08

*P* values (paired *t*-test) are for the difference between ONS and PLACEBO trials. Postprandial concentrations are presented as time-averaged total areas under concentrations vs. time curves. HOMA_IR_ was calculated from fasting insulin and glucose. ISI was calculated from fasting and post-breakfast and post-lunch insulin and glucose according to the Matsuda and DeFronzo model [33]. Mffm/l: insulin sensitivity scores based on fasting insulin and fasting TAG [30].

### Trial order effects

There were no trial order effects on any of the outcomes assessed.

## Discussion

This study found significantly reduced energy intake at the evening meal after high energy ONS was taken in the late afternoon, but a net increase in total energy intake per day over the 5-day supplementation period. On the sixth day ONS had no impact on energy intake and the postprandial responses of subjective appetite, gut appetite hormones and energy expenditure measured under controlled experimental conditions, but fasting plasma insulin concentration and HOMA_IR_ were significantly higher, and insulin sensitivity score based on fasting insulin and TAG significantly lower in the ONS trial. Plasma fasting and postprandial concentrations of glucose and lipids and insulin sensitivity index post-breakfast and lunch were not different between ONS and PLACEBO trials.

It is known that there is some displacement of food from meals during ONS intake [33] but it is not clear whether the timing of intake affects this. Our previous similar study showed that 54% of the energy taken in ONS in the morning, was compensated at the next meal [12], while in this experiment only 43% were compensated, lending some support to the notion that energy intake in the evening might be less satiating than energy intake in the morning [14]. This requires confirmation from a study that investigates the impact of ONS taken both in the morning and the late afternoon by the same participants.

In our previous [12] and this study the reduction in energy intake was observed only at the next meals suggesting that the suppressive action of high energy ONS is short-lived. This is further supported by the finding that energy intake on an experimental day, after five days of supplementation, was not different between the ONS and the PLACEBO trials. It is thus unsurprising that after five days of supplementation there were also no differences in self-rated appetite or its biomarkers [34, 35]. This is consistent with a study in mildly overweight adults, where there were no changes in appetite in the morning after a bedtime snack [15]. Based on the evidence that following ONS intake prior to breakfast, the concentrations of these satiety hormones remained elevated for a duration of 240 minutes [12], the possibility of an acute increase in these hormones in the afternoon after ONS intake cannot be ruled out. This compensatory reduction in energy intake is likely to limit the efficacy of ONS [33], but this should still represent a meaningful increase in energy intake over the five days of supplementation [36]. Despite this, we found no difference in body weight and fat mass changes between the two trials. However, the total energy surplus would be expected to lead to an average increase in body weight of only 0.17 kg (mean cost of weight gain = 31.6 KJ/g (7.55 kcal/g)) [37] and the study was not powered to detect a change of this order.

An energy surplus also seems likely since after ONS fasting plasma insulin concentration and HOMA_IR_, were significantly higher as in other short-term overfeeding studies [22, 23] and insulin sensitivity score based on fasting insulin and fasting TAG was significantly lower. In addition, ISI calculated from fasting and postprandial plasma glucose and insulin concentrations tended (*P* = 0.08) to be lower in the ONS trial. Other studies have reported that sequential meals impinge insulin sensitivity [38], while our study found that in the case of both trials ISI for post-lunch period was not higher than ISI for post-breakfast. This might be related to energy and particular CHO intake being lower in lunch than breakfast meals. It remains to be investigated whether the observed changes in markers of insulin sensitivity persists when ONS are taken for a longer duration. On the other hand, we found no difference in fasting concentrations of glucose, TAG, total-, and LDL-cholesterol concentrations or postprandial responses of glucose, insulin, and TAG.

A strength of this study is an investigation of the use of ONS in free-living individuals, that allowed us to measure energy consumption during meals eaten at home with full-time flexibility for meals, while compliance to the intervention was maintained by daily attendance to the laboratory where participants consumed both ONS and PLACEBO supplements in the presence of the researchers. This study was designed to ensure that participants would not be aware whether they were taking the ONS or the PLACEBO and this is endorsed by the fact that only 4 participants were able to correctly guess about the type of drink they were given. Reassuringly the energy intake of participants who guessed correctly was not different from those who did not. In addition, there was no interaction between the order of the trials on any of the outcomes. In free-living conditions, energy intake may be under- and over-reported [39] but in our study, the energy intake measured in free-living conditions during supplementation with PLACEBO was only slightly lower than energy intake measured under controlled laboratory conditions in the PLACEBO trial.

This study has some limitations. First, the participants were women with no underlying clinical problems regardless of lower BMI and mean FMI (3.17 kg/m^2^) being below the reference range for FMI (3.5–8.7 kg/m^2^) in women of corresponding age [40, 41]. Although we did not screen our participants for eating disorders, none of the participants had menstrual dysfunction, a common feature of eating disorders [42] and their habitual energy intake, measured during the PLACEBO trial and during day 6 of both trials, was typical for women free of eating disorders [43]. Thus, it cannot be assumed that the results are applicable to patients with disease-related malnutrition or eating disorders. Another limitation is that in clinical practice the energy (600 kcal) provided by a single dose of ONS would usually be split into at least two separate “doses” throughout the day.

Coefficients of variance for test-retest repeatability of impedance and body weight were not formally recorded, but in other similar studies, these were only between 3 and 5%. As the measurements of insulin and appetite hormones were expensive, the plasma concentrations were measured only in a subset of 12 randomly selected participants. This decision was based on the previous studies [12, 44] in which plasma concentrations of these hormones were measured in similar numbers. While the study was powered sufficiently to detect the energy intake difference we observed, it may not have been adequately powered to detect other differences, particularly in outcomes measured in only 12 individuals.

Arterial measurements best represent the exposure of peripheral tissues to the hormones and metabolites [45, 46] and arterialized rather than venous blood may have provided a better chance of detecting small differences in appetite hormone concentrations between two trials, but this was not logically or ethically possible. Since venous blood GPL-1 [46], glucose [47] and TAG [48] are lower than in the arterial blood, comparison with studies that used arterial or arterialized blood should be done with care. Finally, it is still unclear whether longer-term supplementation would result in similar responses. Ideally, future studies should investigate longer supplementation in undernourished or sick participants using a randomised controlled design.

In conclusion, consumption of ONS late in the afternoon by free-living healthy women with lower BMI increases total energy intake but has a detrimental impact on measures of insulin sensitivity. Reduced consumption at the meal following supplementation occurs, but this effect is not sustained until the next day. Future research should investigate whether these findings are applicable for patients suffering from disease-related malnutrition or eating disorders and consider the long term effects of ONS.

## Supplementary information

Supplementary Information

## References

[1] NASA Aziz, NIMF Teng, Hamid MRA, Ismail NH (2017). Assessing the nutritional status of hospitalized elderly. Clin Inter Aging.

[2] Avelino-Silva TJ, Jaluul O (2017). Malnutrition in hospitalized older patients: management strategies to improve patient care and clinical outcomes. Int J Gerontol.

[3] Crichton M, Craven D, Mackay H, Marx W, de van der Schueren M, Marshall SA (2018). Systematic review, meta-analysis and meta-regression of the prevalence of protein-energy malnutrition: Associations with geographical region and sex. Age Ageing.

[4] Stratton RJ, Hebuterne X, Elia M (2013). A systematic review and meta-analysis of the impact of oral nutritional supplements on hospital readmissions. Ageing Res Rev.

[5] Bally MR, Yildirim PZB, Bounoure L, Gloy VL, Mueller B, Briel M (2016). Nutritional support and outcomes in malnourished medical inpatients: a systematic review and meta-analysis. JAMA Intern Med..

[6] Elia M, Normand C, Laviano A, Norman K (2016). A systematic review of the cost and cost effectiveness of using standard oral nutritional supplements in community and care home settings. Clin Nutr.

[7] Parsons EL, Stratton RJ, Cawood AL, Smith TR, Elia M (2017). Oral nutritional supplements in a randomised trial are more effective than dietary advice at improving quality of life in malnourished care home residents. Clin Nutr.

[8] Poustie VJ, Russell JE, Watling RM, Ashby D, Smyth RL (2006). Oral protein energy supplements for children with cystic fibrosis:CALICO multicentre randomised controlled trial. BMJ.

[9] Milne AC, Avenell A, Potter J. Oral protein and energy supplementation in older people: A systematic review of randomized trials. In: Home Care Enteral Feeding, vol. 10. Karger Publishers, 2005, pp. 103–25. 10.1159/000083301 15818025

[10] Singh F, Bernstein M, Ryan A, O’neill E, Clements K, Evans WJ (2000). The effect of oral nutritional supplements on habitual dietary quality and quantity in frail elders. J Nutr Health Aging.

[11] Liljeberg E, Andersson A, Blom Malmberg K, Nydahl M (2019). High adherence to oral nutrition supplements prescribed by dietitians: A cross‐sectional study on hospital outpatients. Nutr Clin Pr.

[12] Fatima S, Gerasimidis K, Wright C, Tsiountsioura M, Arvanitidou E-I, Malkova D (2015). Response of appetite and potential appetite regulators following intake of high energy nutritional supplements. Appetite.

[13] Chang CY, Trehan I, Wang RJ, Thakwalakwa C, Maleta K, Deitchler M (2013). Children successfully treated for moderate acute malnutrition remain at risk for malnutrition and death in the subsequent year after recovery. J Nutr.

[14] De Castro JM (2007). The time of day and the proportions of macronutrients eaten are related to total daily food intake. Br J Nutr.

[15] Lay AH, Crabtree DR, Campbell TG, Dreczkowski GM, Galloway SD, Tipton KD (2018). A bedtime milk snack does not impact rmr, substrate utilisation and appetite the following morning in mildly overweight males. Br J Nutr.

[16] Kinsey AW, Ormsbee MJ (2015). The health impact of nighttime eating: old and new perspectives. Nutrients.

[17] Hopkins M, Beaulieu K, Myers A, Gibbons C, Blundell JE (2017). Mechanisms responsible for homeostatic appetite control: theoretical advances and practical implications. Expert Rev Endocrinol Metab.

[18] Lean M, Malkova D (2016). Altered gut and adipose tissue hormones in overweight and obese individuals: cause or consequence?. Int J Obes.

[19] Doucet É, Laviolette M, Imbeault P, Strychar I, Rabasa-Lhoret R, Prud’homme D (2008). Total peptide YY is a correlate of postprandial energy expenditure but not of appetite or energy intake in healthy women. Metabolism.

[20] De Graaf C, Blom WA, Smeets PA, Stafleu A, Hendriks HF (2004). Biomarkers of satiation and satiety. Am J Clin Nutr.

[21] Karl JP, Young AJ, Rood JC, Montain SJ (2013). Independent and combined effects of eating rate and energy density on energy intake, appetite, and gut hormones. Obesity.

[22] Smith GI, Magkos F, Reeds DN, Okunade AL, Patterson BW, Mittendorfer B (2013). One day of mixed meal overfeeding reduces hepatic insulin sensitivity and increases VLDL particle but not VLDL-triglyceride secretion in overweight and obese men. J Clin Endocrinol Metab.

[23] Parry SA, Smith JR, Corbett TR, Woods RM, Hulston CJ (2017). Short-term, high-fat overfeeding impairs glycaemic control but does not alter gut hormone responses to a mixed meal tolerance test in healthy, normal-weight individuals. Br J Nutr.

[24] Guthold R, Stevens GA, Riley LM, Bull FC (2018). Worldwide trends in insufficient physical activity from 2001 to 2016: A pooled analysis of 358 population-based surveys with 1· 9 million participants. Lancet Glob Health..

[25] Manthou E, Gill JM, Malkova D (2015). Effect of exercise programs with aerobic exercise sessions of similar intensity but different frequency and duration on health-related measures in overweight women. J Phys Act Health.

[26] de Castro JM (2000). Eating behavior: Lessons from the real world of humans. Nutrition.

[27] Goldberg G, Black A, Jebb S, Cole T, Murgatroyd P, Coward W (1991). Critical evaluation of energy intake data using fundamental principles of energy physiology: 1. Derivation of cut-off limits to identify under-recording. Eur J Clin Nutr.

[28] Flint A, Raben A, Blundell JE, Astrup A (2000). Reproducibility, power and validity of visual analogue scales in assessment of appetite sensations in single test meal studies. Int J Obes Relat Metab Disord.

[29] Matthews D, Hosker J, Rudenski A, Naylor B, Treacher D, Turner R (1985). Homeostasis model assessment: insulin resistance and β-cell function from fasting plasma glucose and insulin concentrations in man. Diabetologia.

[30] McAuley KA, Williams SM, Mann JI, Walker RJ, Lewis-Barned NJ, Temple LA (2001). Diagnosing insulin resistance in the general population. Diabetes Care.

[31] Maki KC, Rains TM, Dicklin MR, Bell M (2010). Repeatability of indices of insulin sensitivity and secretion from standard liquid meal tests in subjects with type 2 diabetes mellitus or normal or impaired fasting glucose. Diabetes Technol Therapeutics.

[32] Matsuda M, DeFronzo RA (1999). Insulin sensitivity indices obtained from oral glucose tolerance testing: Comparison with the euglycemic insulin clamp. Diabetes care.

[33] Fiatarone MS, Bernstein M, Ryan A, O’neill E, Clements K, Evans WJ (2000). The effect of oral nutritional supplements on habitual dietary quality and quantity in frail elders. J Nutr Health Aging.

[34] Sobrino Crespo C, Perianes Cachero A, Puebla Jiménez L, Barrios V, Arilla Ferreiro E (2014). Peptides and food intake. Front Endocrinol.

[35] Huda M, Wilding J, Pinkney J (2006). Gut peptides and the regulation of appetite. Obes Rev.

[36] Reinders I, Volkert D, de Groot LC, Beck AM, Feldblum I, Jobse I (2019). Effectiveness of nutritional interventions in older adults at risk of malnutrition across different health care settings: Pooled analyses of individual participant data from nine randomized controlled trials. Clin Nutr.

[37] Forbes GB, Brown MR, Welle SL, Lipinski BA (1986). Deliberate overfeeding in women and men: Energy cost and composition of the weight gain. Br J Nutr.

[38] Ping-Delfos WCS, Soares M (2011). Diet induced thermogenesis, fat oxidation and food intake following sequential meals: influence of calcium and vitamin D. Clin Nutr.

[39] Johansson L, Solvoll K, Bjørneboe G-E, Drevon CA (1998). Under-and overreporting of energy intake related to weight status and lifestyle in a nationwide sample. Am J Clin Nutr.

[40] Schutz Y, Kyle U, Pichard C (2002). Fat-free mass index and fat mass index percentiles in Caucasians aged 18–98 y. Int J Obes.

[41] Kyle UG, Schutz Y, Dupertuis YM, Pichard C (2003). Body composition interpretation: Contributions of the fat-free mass index and the body fat mass index. Nutrition.

[42] Boisseau CL (2016). Identification and management of eating disorders in gynecology: Menstrual health as an underutilized screening tool. Am J Obstet Gynecol.

[43] Hadigan CM, Anderson EJ, Miller KK, Hubbard JL, Herzog DB, Klibanski A (2000). Assessment of macronutrient and micronutrient intake in women with anorexia nervosa. Int J Eat Disord.

[44] Alfheeaid H, Gerasimidis K, Năstase A-M, Elhauge M, Cochrane B, Malkova D (2018). Impact of phenylketonuria type meal on appetite, thermic effect of feeding and postprandial fat oxidation. Clin Nutr.

[45] Liu D, Moberg E, Kollind M, Lins P-E, Adamson U, Macdonald I (1992). Arterial, arterialized venous, venous and capillary blood glucose measurements in normal man during hyperinsulinaemic euglycaemia and hypoglycaemia. Diabetologia.

[46] Chen YC, Edinburgh RM, Hengist A, Smith HA, Walhin JP, Betts JA (2018). Venous blood provides lower glucagon-like peptide-1 concentrations than arterialized blood in the postprandial but not the fasted state: Consequences of sampling methods. Exp Physiol.

[47] Edinburgh RM, Hengist A, Smith HA, Betts JA, Thompson D, Walhin J-P (2017). Prior exercise alters the difference between arterialised and venous glycaemia: Implications for blood sampling procedures. Br J Nutr.

[48] Malkova D, Evans R, Frayn K, Humphreys S, Jones P, Hardman A (2000). Prior exercise and postprandial substrate extraction across the human leg. Am J Physiol. Endocrinol Metab.

