# Supplementary material for: Impact of high energy oral nutritional supplements consumed in the late afternoon on appetite, energy intake and cardio-metabolic risk factors in females with lower BMI
Source: Eur J Clin Nutr. 2021 Nov 12;76(6):811–8. doi: 10.1038/s41430-021-01042-w (PMC9187517; doi:10.1038/s41430-021-01042-w)
Supplement: Supplementary file 1 — Supplementary Information [file 41430_2021_1042_MOESM1_ESM.docx]

**Impact of High Energy Oral Nutritional Supplements Consumed in the Late Afternoon on Appetite, Energy Intake and Cardio-Metabolic Risk Factors in Females with lower BMI: Supplementary Material**

Sadia Fatima^a,b^, Konstantinos Gerasimidis^a^, Charlotte Wright^a^, Dalia Malkova^a*^

^a^ Human Nutrition, School of Medicine, Dentistry and Nursing, College of Medical, Veterinary and Life Sciences, University of Glasgow, Glasgow, UK; ^b^Khyber Medical University Peshawar, Pakistan

**Supplementary Methods**

*Composition of meals*

Breakfast included a variety of breakfast cereals (corn flakes, fruit & fibre or coco pops), milk (semi-skimmed, skimmed and whole cream), croissants, jam (apricot, mixed fruit jam, black-current), butter, juice (apple and orange) and fruits (apple, banana, pineapple and grapes). Lunch included two white or whole meal bread sandwiches, a mixed leaf salad, yogurt (plain, flavoured), fruits (apple, banana, grapes) and juice (apple, orange, and blackcurrant). For dinner, participants selected the meal of their choice from the cafeteria of the local hospital, which was then served with fruits (apple, banana, grapes and pineapple), yogurt (plain, flavoured) and juice (apple, orange, and blackcurrant).

*Energy Expenditure*

Rates of oxygen consumption (V̇O_2_) and carbon dioxide production (V̇CO_2_) were measured by computerised open-circuit ventilated hood system (Oxycon Pro, Jaeger GmbH, Germany). Participants lay supine, still and awake during the measurement. Once comfortable, a clear plastic canopy (weight, 550 g; dimensions, 19.6 x 12.99 x 9.44 inch) was placed on the participant’s head and expired gas was collected. Records of V̇O_2_ and V̇CO_2_ were obtained every 30 seconds and averaged prior to calculations of energy expenditure rate using indirect calorimetry equations. For the validation of the accuracy of Oxycon Pro system, an alcohol-burning test was conducted weekly with a CV of 1.6%. Volume and gas calibrations were performed prior to each measurement and were accepted if differences were ≤ ±1%.

*Plasma Preparation and Blood Analysis*

Venous blood samples were collected into EDTA tubes (BD Vacutainer Systems, Plymouth, UK). Plasma used for the analysis of lipid, insulin and glucose concentrations was prepared by blood centrifugation at 4^0^C, 3000 rpm for 15mins. Blood samples used for analysis of GLP-1 and PYY concentrations was treated with Aprotinin (0.6 TIU activity per ml, Sigma-Aldrich, UK) and then centrifuged at 3000 rpm for 10 minutes. The plasma spares were kept at -70^0^C until analysis. As the analysis of appetite hormones was expensive, the responses of plasma concentrations of PYY and active GLP were measured in only a subset of 12 randomly selected participants. Commercially available ELISA kits were used to measure plasma concentration of total PYY (Merck, Millipore, Bioscience Division, UK), active GLP-1 (Merck, Millipore, Bioscience Division, UK), and insulin (Mercodia AB, Uppsala, Sweden). Plasma concentrations of total-and HDL-cholesterol (ABX Pentra, Horiba, France), TAG (ABX Pentra, Horiba, France), and glucose (Glucose HK CP Reagent ABX Pentra, Horiba, France) were measured by colorimetric methods. The within-batch coefficients of variation were <3% for plasma lipids and glucose, <4% for the insulin and, <8% for the activated GLP-1 and PYY assays.

**Supplementary Table** **1**. Energy, carbohydrate (CHO), fat and protein provided by the PLACEBO and the high energy oral nutritional supplement (ONS).

|  | PLACEBO | ONS |
| --- | --- | --- |
| CHO (g) | 11.3 | 68.8 |
| Fat (g) | 1.3 | 30.4 |
| Protein (g) | 9 | 11.9 |
| Energy (MJ) | 0.38 | 2.49 |
| Energy from CHO (%) | 48 | 46 |
| Energy from Fat (%) | 13 | 46 |
| Energy from Protein (%) | 39 | 8 |

**Supplementary Figure** **1**. Fasting (0 min) and postprandial (0-270 min) responses of metabolic rate measured on the day following 5 days of supplementation with ONS and PLACEBO. *Ad libitum* breakfast and *ad libitum* lunch were provided after fasting measurements and 120 minutes after that, respectively. Values are presented as Mean ± SE (n=22).

**Supplementary Figure 2**. Fasting (0 min) and postprandial (0-270 min) responses of glucose, insulin triglycerides (TAG) measured on the day following 5 days of supplementation with ONS and PLACEBO. *Ad libitum* breakfast and *ad libitum* lunch were provided after fasting measurements and 120 minutes after that, respectively. Values are presented as Means ± SE (n=12). Responses were analysed by two-way repeated measures ANOVA.
